# Biological injection therapy with leukocyte-poor platelet-rich plasma induces cellular alterations, enhancement of lubricin, and inflammatory downregulation *in vivo* in human knees: A controlled, prospective human clinical trial based on mass spectrometry imaging analysis

**DOI:** 10.3389/fsurg.2023.1169112

**Published:** 2023-04-21

**Authors:** Axel W. Baltzer, Rita Casadonte, Alexei Korff, Lea Merline Baltzer, Katharina Kriegsmann, Mark Kriegsmann, Jörg Kriegsmann

**Affiliations:** ^1^ Center for Molecular Orthopaedics, MVZ Ortho Koenigsallee, Düsseldorf, Germany; ^2^ Imaging Mass Spectrometry, Proteopath GmbH, Trier, Germany; ^3^ Klinik für Zahnmedizin, Uniklinik RWTH, Aachen, Germany; ^4^ Department for Hematology, Oncology and Rheumatology, University of Heidelberg, Heidelberg, Germany; ^5^ Institute of Pathology, University Hospital Heidelberg, Heidelberg, Germany; ^6^ Germany Translational Lung Research Centre Heidelberg, Member of the German Centre for Lung Research (DZL), Heidelberg, Germany; ^7^ MVZ-Zentrum für Histologie, Zytologie und Molekulare Diagnostik, Trier, Germany; ^8^ Department of Medicine, Faculty of Medicine/Dentistry, Danube Private University, Krems, Austria

**Keywords:** disease-modifying therapy, inflammation, LpPRP, MALDI-mass spectrometry, osteoarthritis, platelet-rich plasma, lubricin

## Abstract

**Objective:**

To investigate the *in vivo* biological effects of leukocyte-poor platelet-rich plasma (LpPRP) treatment in human synovial layer to establish the cellular basis for a prolonged clinical improvement.

**Methods:**

Synovial tissues (*n* = 367) were prospectively collected from patients undergoing arthroscopic surgery. Autologous-conditioned plasma, LpPRP, was injected into the knees of 163 patients 1–7 days before surgery to reduce operative trauma and inflammation, and to induce the onset of regeneration. A total of 204 patients did not receive any injection. All samples were analyzed by mass spectrometry imaging. Data analysis was evaluated by clustering, classification, and investigation of predictive peptides. Peptide identification was done by tandem mass spectrometry and database matching.

**Results:**

Data analysis revealed two major clusters belonging to LpPRP-treated (LpPRP-1) and untreated (LpPRP-0) patients. Classification analysis showed a discrimination accuracy of 82%–90%. We identified discriminating peptides for CD45 and CD29 receptors (receptor-type tyrosine-protein phosphatase C and integrin beta 1), indicating an enhancement of musculoskeletal stem cells, as well as an enhancement of lubricin, collagen alpha-1-(I) chain, and interleukin-receptor-17-E, dampening the inflammatory reaction in the LpPRP-1 group following LpPRP injection.

**Conclusions:**

We could demonstrate for the first time that injection therapy using “autologic-conditioned biologics” may lead to cellular changes in the synovial membrane that might explain the reported prolonged beneficial clinical effects. Here, we show *in vivo* cellular changes, possibly based on muscular skeletal stem cell alterations, in the synovial layer. The gliding capacities of joints might be improved by enhancing of lubricin, anti-inflammation by activation of interleukin-17 receptor E, and reduction of the inflammatory process by blocking interleukin-17.

## 1. Introduction

For about 20 years, growth factors (GFs) and autologous-conditioned biologics (ACBs), such as platelet-rich plasma (PRP), have been clinically used in our institution (1, 2) and investigated by others for the treatment of clinical symptoms such as pain, stiffness, and reduced activities of daily living (ADLs) in inflammatory and degenerative joint diseases such as osteoarthritis (OA) (3). In the last few years, PRP therapies have become increasingly important and are now available almost worldwide. This fact supports the therapeutic success of ACBs in orthopedics, not least for the treatment of different grades of OA, one of the main goals of orthopedic surgeons (1, 2, 4–6).

The outcome of PRP therapy in different joints has been investigated by several studies, most of which affirm a better effect than corticosteroids or hyaluronic acid (HA) in terms of the duration of the positive therapeutic effects over time. Results of studies reported in the literature indicate up to 75% reduction in pain for patients treated with PRP. One major advantage is the prolonged period of pain relief, improved range of motion (ROM), and ADLs in these patients. It has been described that the positive effect after treatment lasts up to 12 months (4, 6–8).

In his 2020 review, DePhillipo reports that there is a predominantly higher number of clinical trials investigating symptomatic resolution therapy in OA (89%) compared with only 5% preventative therapies and 6% disease-modifying therapies (9). Some studies have investigated synovial fluid consumption after PRP treatment, comparing it with that of untreated or HA-treated patients. Elevated levels of anti-inflammatory cytokines or regenerative growth factors were detected up to 12 weeks after PRP injection into the joints (6, 7, 10–13). To the best of our knowledge, the possible cellular changes that lead to this longer-lasting positive results of PRP have never been investigated in *in vivo* human clinical studies analyzing treated tissues (14). PRP therapy is a purely biological, autologous injection treatment based on a concentration of GF and cytokines produced by the stimulation of white blood cells and activation of platelets by a centrifugation process of the individual's whole blood, and subsequent collection of these and platelets in plasma. Many providers sell different systems to produce different PRPs, but in general they can be divided into two major PRP groups: leukocyte-rich PRP (LrPRP) and leukocyte-poor PRP (LpPRP). Different treatment results and theories about different underlying cellular mechanisms have been discussed in the literature, but *in vivo* human clinical studies to evaluate cellular changes within the synovial fluid and inflammatory response after PRP treatment have not yet been performed or published (9, 15–18).

The purpose of this study is to analyze cellular changes in the human synovial layer 3 days after injection with LpPRP, to establish the cellular basis to be correlated with long term clinical improvement. To assess cellular changes, we used a matrix-assisted laser desorption/ionization (MALDI) mass spectrometry imaging (MSI) technique that combines the investigation of hundreds of peptides with information about their distribution in tissue. This method has been used previously in non-neoplastic and neoplastic diseases and is suitable to provide meaningful information on cellular changes in human diseases (19–21). We hypothesize that cellular changes at the synovial level in OA joints from an activated inflammatory environment to an anti-inflammatory environment may account for the prolonged clinical improvement after LpPRP injections, chemotaxis, proliferation, and differentiation of stem cells driven by persistently altered cellular expression patterns of cytokines and growth factors. These alterations underlie regeneration and the anti-inflammatory process (22).

## 2. Materials and methods

### 2.1. Sample collection

This study was conducted to investigate, by histological and mass spectrometry procedures, the anti-inflammatory and cellular regenerative changes on the synovial layer of knee joints following injection of LpPRP (Arthrex) before surgery. The effect of growth factors is generated by binding to cell surface receptors within minutes or hours. This moderates the expression patterns of the cells that have been contacted. To study the effects on cells after the completion of this moderation process, which are established at the latest after 1 day, LpPRP was administered 1–7 days prior to surgery. Intra-articular injection of 3 ml of LpPRP was performed *via* the anterolateral approach while the patient was in a sitting position with the leg dangling. The type of administration and dose of the LpPRP was always the same in all treated patients. Synovial tissue samples were collected from patients undergoing arthroscopic surgery due to meniscal tears or focal degenerative cartilage instabilities in the period 2015–2020. All patients received partial meniscectomy and/or chondral abrasion arthroplasty, and partial synovectomy. Eligible patients were all Eurasians, healthy and informed adults. Patients with known joint diseases such as rheumatoid arthritis or posttraumatic osteoarthritis (PTOA) were excluded. A total of 367 patients were included in this study, of whom 163 were treated with LpPRP (LpPRP-1) and 204 patients did not receive LpPRP injection (LpPRP-0) before surgery. Patient's characteristics are described in Supplementary Table S1, Figure S1. The study design is prospective, observer-blinded, and randomized. The decision to give the injection prior to surgery was independent of the investigator and the clinical status of the patients, but strictly dependent on the patients’ willingness and attitude to receive a preoperative LpPRP injection.

This study was approved by the Ethics Committee of the Heinrich-Heine-University Duesseldorf (#4762), and all patients gave a written consent. This study did not receive any financial support.

### 2.2. Sample processing

Synovial tissue samples were taken from the operated joints and immediately fixed in 10% neutral buffered formalin for 12–24 h. Samples were subsequently processed to create paraffin-embedded specimens and further analyzed for histopathological examination. In order to facilitate the molecular analysis of the large number of the synovial samples, multiple formalin-fixed paraffin-embedded (FFPE) synovial tissues were assembled in a single FFPE block to form a tissue microarray (TMA) using an automatic TMA instrument (TMA Grand Master, 3DHistech, Budapest, Hungary). Before TMA construction, photomicrographs of hematoxylin and eosin (HE)-stained sections were examined and digitally marked by a pathologist (JK) over the synovial lining layer. Subsequently, small cylindrical tissue cores (1 mm in diameter) were punched from the specific annotated region of interest within each FFPE “donor” block and transferred in an empty recipient paraffin block with preformed holes in an array-like format and equally spaced 0.5 mm apart (20). A total of seven TMAs were then generated with six TMAs including each 116 tissue cores (58 different patients in duplicate), and one TMA including 38 tissue cores (19 different patients in duplicate). The layout of each TMA included both LpPRP-1 and LpPRP-0 tissue types and at least two tissue core punches from the same patient. In addition, four muscle core samples, collected from human leg skeletal muscle tissues, were incorporated in each TMA, which were used as internal experimental control to assure standardization of parameters during sample preparation so that inter/intra-assay variations are minimized. From each TMA, two serial sections (3 µm thick) were collected, from which one was mounted onto conductive indium tin oxide (ITO)-coated glass slides (Bruker Daltonik, Bremen, Germany), pretreated with 0.1% (v/v in water) of poly-L-lysine solution (Sigma Aldrich Chemie, Taufkirchen, Germany), dried overnight at 37°C, and then stored at room temperature until MALDI MSI analysis. The other section was stained by HE for histopathological examination.

### 2.3. Preparation for MALDI sample MSI analysis

A sample preparation protocol for MALDI analysis of FFPE tissues was previously established (23) and here modified following the procedure: (i) heating FFPE TMA slides at 80°C for melting paraffin; (ii) tissue dewaxing in 100% xylene (twice for 5 min), and washing in 100% isopropanol (5 min), graded ethanol baths (100%, 95%, 70%, 50%), and deionized water each for 5 min; (iii) antigen retrieval in 10 mM Tris buffer (Sigma Aldrich Chemie) pH = 9, at 95°C for 20 min using a decloaking chamber (ZITOMED Systems GmbH, Berlin, Germany); (iv) deposition of 0.03 mg/ml trypsin (Promega, Mannheim, Germany) solution in 20 mM ammonium bicarbonate (Sigma Aldrich Chemie) using an automatic reagent sprayer (TM-Sprayer, HTX Technologies, Chapel Hill, NC, USA); (v) *in situ* digestion in a humidity chamber at 50°C for 2 h; (vi) deposition of α-cyano-4-hydroxycinnamic acid matrix (CHCA, Bruker Daltonik, Bremen, Germany) solution (10 mg/ml in 70% acetonitrile, 1% trifluoroacetic acid) using the same TM-Sprayer. Trypsin and matrix deposition methods were reported previously (23, 24) and are reproduced here in Supplementary Table S2. All solvents and trifluoroacetic acid are acquired from Fisher Scientific (Schwerte, Germany).

### 2.4. MALDI MSI analysis

Mass spectrometry (MS) measurements are carried out using a rapifleX MALDI Tissuetyper (Bruker Daltonik) controlled by the FlexControl 4.2 software package, operated in the positive reflector mode on a mass/charge (*m*/*z*) range 600–3,200. A customized raster of spot arrays is created to analyze the entire TMAs with 20 μm center-to-center spacing between each spot using FlexImaging 6.0 (Bruker Daltonik) software package. Totally, 200 laser shots are acquired from each tissue spot at a laser frequency of 10 kHz with single Smartbeam Parameter at 20 µm × 20 µm scan range. The MS method is externally calibrated using the peptide calibration standard II (Bruker Daltonik). All mass spectra are baseline subtracted during acquisition with the TopHat algorithm. After MALDI analysis, the matrix is removed in 2× 100% ethanol washes and HE staining is performed. Slides are scanned with 40× objective magnification using two automated slide scanners (Aperio AT2 slide scanner, Leica Biosystems, Wetzlar, Germany, and 3DHISTECH Ltd., Budapest, Hungary), and regions of interest are annotated by an expert pathologist for data analysis.

### 2.5. Protein identification

Tissue sections are *in situ* digested with trypsin and sprayed with CHCA matrix solution following the same procedure described above. The *m*/*z* species peptides, with expressions that are found to be significantly different between LpPRP-0 and LpPRP-1, are identified using a rapifleX MALDI Tissue-type mass spectrometer (Bruker Daltonik) in the tandem mass spectrometry (MS/MS) mode that provided amino acid sequence information from specific peptide fragments. In this approach, the peptide precursor of interest is selected and fragmented in a collision cell using nitrogen as a collision gas. The resulting spectrum fragmentation patterns are searched against the MASCOT database (Mascot Server version 2.8.0, SwissProt: SwissProt 2021_04.fasta) for matching the tryptic peptide sequences to their corresponding intact proteins. The MS/MS spectrum search parameters included MS tolerance of ±200 ppm, MS/MS tolerance of ±0.5 Da, 1 missed cleavage, and the following variable modifications: acetyl (protein N-term), oxidation (HW), oxidation (M), and oxidation (P).

### 2.6. Data analysis

SCiLS Lab (Bruker Daltonik) and R-statistical software (25) (version 3.5.3 and 4.0.3) are used for data preprocessing, which included total ion count normalization and mass alignment (26), and classification analysis. Principal component analysis (PCA) is performed using SCiLS Lab for visual exploration of the main variance on the whole dataset. The dataset comprising hundreds or thousands of variables is then simplified by reducing the variables to the main components. The Uniform Manifold Approximation and Projection (UMAP) analysis is performed as an alternative dimension reduction for visualizing clusters between the two sample types LpPRP-0 and LpPRP-1. Mass spectral data from annotated regions are used for classification *via* linear discriminant analysis (LDA) and support vector machine (SVM) algorithms. The robustness of the machine learning models on the spectra and patient level is estimated with F1 score and two cross-validation methods, leave-one-out cross-validation (LOOCV) and *k*-fold. In the latter case, the dataset is randomly split in *k* = 10 subgroups of samples (spectra or patients), each including both LpPRP-0 and LpPRP-1 samples. Thus, 10 cross-validation folds are performed where each group is given the opportunity to be used as a test set while all other groups together are used as a training dataset. LDA and SVM models are also evaluated based on the most relevant spectral features (*m*/*z* ion peptides) to enhance the classification performance. These *m*/*z* features are selected either by a receiver operating characteristic (ROC) analysis or by a stepwise forward feature selection. At last, the best relevant features are selected one by one until the increase in accuracy fell under the specified threshold (0.001).

## 3. Results

### 3.1. Patients’ analysis

All HE-stained tissue microarrays were histologically examined under light microscopy by a pathologist (JK) after MSI analysis, who marked synovial lining layer regions with inflammation. Some tissues could not be annotated because they were too small, or tissue cores were lost or folded in the TMAs during the staining procedure. Therefore, 289 of 367 patients were used for the classification analysis, where 128 patients were treated with LpPRP (LpPRP-1) and 161 patients did not have LpPRP treatment. The histological annotated tissue sections were then co-registered with the image of the same section analyzed by MSI, enabling individual mass spectra obtained from each coordinate position on the tissue to be linked to that same specific histological region in the tissue microarray.

The two groups selected for classification analysis were comparable with a mean age in the LpPRP-1 group of 53.8 years (SD = 9.8), compared to 54.6 years (SD = 9.8) in the LpPRP-0 group. The male/female ratio in the LpPRP-1 group was 1:1.09, while it was 1:0.96 in the LpPRP-0 group. Average time between LpPRP injection and surgery in the LpPRP-1 group was 3.026 days, with a range of 1–7 days. Pain at baseline was evaluated by visual analogue scale (VAS) and was equal in both groups (*p*-value > 0.05) Histopathological examination based on HE staining showed degenerative changes, mild inflammation, and no difference in the extent of synovitis between groups (Supplementary Figure S2).

### 3.2. Classification analysis

**Figure 1:**
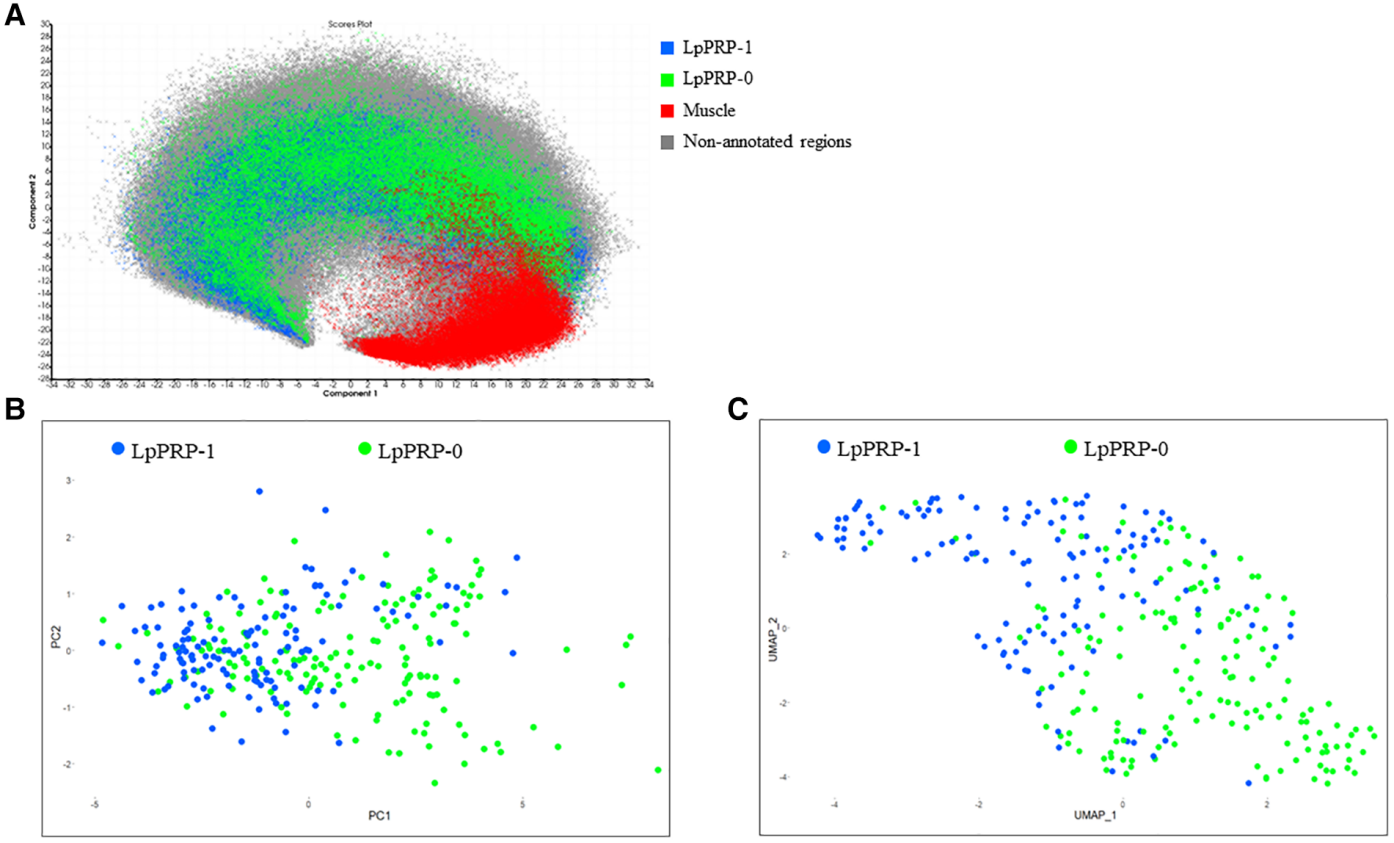
(**A**) Principal component analysis of the whole TMA dataset performed with SCiLS Lab software. Spectra from annotated regions of LpPRP-1 (blue) and LpPRP-0 (green) from all TMAs cluster together and are separated from muscle tissues (red). All muscle spectra (red) from all TMAs cluster in the same group. Each pixel corresponds to 1 spectrum. Principal component analysis (**B**) and UMAP analysis (**C**) of the annotated regions including spectra with *m*/*z* signals having an AUROC ≥ 0.7 show two clusters corresponding to LpPRP-0 (pixels in green) and LpPRP-1 (pixels in blue). PCA and UMAP were performed on patients, thus each pixel corresponds to one patient. UMAP analysis shows better separation of LpPRP-1 and LpPRP-0 patients. UMAP parameters used: n_neighbors = 2, min_dist = 0.3. TMA, tissue microarray; AUROC, area under the ROC curve; PCA, principal component analysis; LpPRP, leukocyte-poor platelet-rich plasma; UMAP, Uniform Manifold Approximation and Projection; ROC, receiver operating characteristic.

In a prior investigation, an unsupervised analysis is performed on the whole dataset including all synovial and muscle control tissues using PCA, which mainly separated synovial from muscle spectra (Figure 1A). This shows that there is no separation between different TMAs that could be related to technical variation (i.e., different experimental conditions due to TMA measurements on different days). This was confirmed by univariate statistical Wilcoxon test that was used to compare spectral data from TMA random groups: TMA group 1 (TMA 2, 3, 4, vs. TMA 5, 6, 7); TMA group 2 (TMA 2, 4, 6 vs. TMA 3, 5, 7). Likewise, Wilcoxon test was applied to compare spectral data from muscle tissues random groups: muscle group 1 (muscles tissues from TMA 2, 3, 4, vs. muscle tissues from TMA 5, 6, 7; muscle group 2 (muscles tissues from TMA 2, 4, 6, vs. muscle tissues from TMA 3, 5, 7) (Supplementary Table S3). In the TMA tests, the mean *p*-values were 0.51 and 0.8 for the TMA groups 1 and 2, whereas in the muscle tissue tests, the mean *p*-values were 0.78 and 0.81, respectively. This indicated that there were no statistically significant differences between these groups. In contrast, Wilcoxon's test performed on spectral data from LpPRP-treated (LpPRP-1) and untreated tissues (LpPRP-0) showed a mean *p*-value = 0, denoting a significant difference (Supplementary Table S3). When only spectra with *m*/*z* having an area under the ROC curve (AUROC) ≥ 0.7 was considered for each patient, PCA showed two clusters of spectra belonging to LpPRP-0 and LpPRP-1, respectively; however, some LpPRP-0 and LpPRP-1 patients resulted in overlapping (Figure 1B). UMAP analysis showed better separation of LpPRP-1 and LpPRP-0 patients (Figure 1C).

Several classification approaches are examined using LDA and SVM algorithms with either LOOCV or *k-*fold (*k* = 10) cross-validations in order to identify molecular differences between treated (LpPRP-1) and nontreated (LpPRP-0) patients. The highest classification accuracy resulted from LDA analysis based on individual spectra followed by major voting, which was 87% with LOOCV and 90% with *k*-fold cross-validation (Table 1A). Other classification strategies, including LDA based on individual spectra (Table 1A), LDA and SVM based on mean spectra for each patient (Table 1B), LDA and SVM based on feature selected by AUROC ≥ 0.7 (number of *m*/*z* features selected = 41) (Table 1C), and LDA and SVM based on the most relevant features selected by forward stepwise selection procedure (Table 1D), achieved comparable performance to discriminate LpPRP-0 from LpPRP-1 patients with an accuracy of 81%–84%. The lowest accuracy was obtained performing classification based on *m*/*z* features selected by AUROC ≥ 0.75 (number of *m*/*z* features selected = 11) with both LDA (78%) and SVM (77%) algorithms (Table 1C). F1 score was calculated for each classification strategy as a metric used to measure the performance of classification models. F1 score can be interpreted as a harmonic mean of the precision and recall, and it is used to evaluate binary classification systems. F1 score is a number that reaches its best value at 1 and worst score at 0 (27). F1 score mean of all classification models was = 0.84, which is considered a good score to assess model performance. In addition, classification results were compared to the results of two different types of random-guess metrics: (1) a “maximum class guess,” where all observations are predicted as belonging to the most abundant class; (2) a “random-weighted-guess” classifier, in which the probability of the classification for each spectrum is defined by the prior distribution in then training set. The results of LDA and SVM models were better (F1 score mean = 0.84, Table 1) than the maximum class guess (score = 0.53) and the random-weighted guess (score = 0.51).

**Table 1:** Classification approaches to discriminate LpPRP-treated and -untreated patients.

(A) Classification based on spectra						
Model	Cross-validation	Tissue type	Correct spectra/total spectra	Accuracy on individual spectra	Accuracy on individual spectra and majority vote*	F1 score
LDA	LOOCV	LpPRP-0	26,934/31,932	85%	87%	0.84
		LpPRP-1	26,587/31,294			
	*k*-fold (*k* = 10)	LpPRP-0	26,725/31,294	84%	90%	0.84
		LpPRP-1	26,677/31,294			
(B) Classification based on patients						
Model		Cross-validation	Tissue type	Correct patient/total patients	Accuracy	F1 score
LDA		LOOCV	LpPRP-0	138/161	83%	0.85
			LpPRP-1	103/128		
		*k-*fold (*k* = 10)	LpPRP-0	133/161	82%	0.84
			LpPRP-1	103/128		
SVM		LOOCV	LpPRP-0	142/161	84%	0.86
			LpPRP-1	102/128		
		*k-*fold (*k* = 10)	LpPRP-0	144/161	85%	0.87
			LpPRP-1	103/128		
(C) Classification based on feature selected by AUROC						
Model	AUROC	Tissue type	Correct patient/total patients	Number of features	Accuracy	F1 score
LDA	≥0.7	LpPRP-0	134/161	41	81%	0.83
		LpPRP-1	101/128			
	≥0.75	LpPRP-0	125/161	11	78%	0.83
		LpPRP-1	101/128			
SVM	≥0.7	LpPRP-0	137/161	41	82%	0.84
		LpPRP-1	100/128			
	≥0.75	LpPRP-0	128/161	11	77%	0.79
		LpPRP-1	96/128			
(D) Classification based on forward feature selection						
Model		Tissue type	Correct patient/total patients	Number of features	Accuracy	F1 score
LDA		LpPRP-0	137/161	9	85%	0.86
		LpPRP-1	109/128			
SVM		LpPRP-0	139/161	9	84%	0.85
		LpPRP-1	103/128			

LpPRP, leukocyte-poor platelet-rich plasma; LDA, linear discriminant analysis; LOOCV, leave-one-out cross-validation; SVM, support vector machine; AUROC, area under the ROC curve.

* Classification based on majority vote: first individual spectra are assigned to a class, then the majority of spectra are assigned for each patient and the percentage of patients correctly classified is calculated.

### 3.3. Protein identification

Comparison of the average relative intensities at each *m*/*z* value between the two sample types of the classification cohort revealed 48 differential *m*/*z* features exhibiting a weight statistical significance (area under the ROC ≥ 0.7, *p*-value ≤ 0.001). Some of those *m*/*z* features could be identified and associated with their respective parent proteins through database matching. Details of the results of identification analyses are reported in Table 2, including results of the MASCOT database research with scores (ion scores > 33 indicate identity or extensive homology, *p* < 0.05) and results from tandem mass spectrometry experiments (peptide sequences and name of the proteins identified). Specifically, 16 out of 48 *m*/*z* peptides are identified by MS/MS directly from the digested tissue, from which, 13 peptides are highly expressed in LpPRP-1 (*m*/*z* 1121.54, 1137.74, 1138.5, 1140.65, 1141.6, 1510.72, 1512.73, 1671.8, 1672.8, 1816.94, 1818.94, 2164.1, and 2303.17), and 3 peptides (*m*/*z* 961.49, 1759.9, and 1850.1) are highly expressed in LpPRP-0 (Table 2, masses indicated with an asterisk). One peptide (*m*/*z* 961.49) is identified as collagen alpha-1(III) chain, one peptide (*m*/*z* 1121.54) is identified as interleukin-1 receptor accessory protein, four peptides (*m*/*z* 1,137.74, 1,510.72, 1,512.73, 1,818.94) are identified as receptor-type tyrosine-protein phosphatase C (CD45), one peptide is identified as collagen alpha-2(IX) chain (*m*/*z* 1,138.5), two peptides are identified as proteoglycan 4 (lubricin) (*m*/*z* 1,141.6, 2,303.17), one peptide is identified as integrin beta-1 (CD29) (*m*/*z* 1,140.65), two peptides are identified as interleukin-17 receptor E (*m*/*z* 1,671.8, 2,164.1), two peptides are identified as collagen alpha-1(I) chain precursor (*m*/*z* 1,672.8, 1,816.94), one peptide is identified as collagen alpha-1(II) chain (*m*/*z* 1,759.9), and one peptide is identified as protein S100-A11 (calgizzarin) (*m*/*z* 1,850.1). The MASCOT score was ≥67 and ≤145, only two peptide sequences are identified with a low mascot score of 37 for the collagen alpha-1(III) chain and 38 for the collagen alpha-1(I) chain precursor. Representative fragmented peptides related to collagen alpha-1(III) chain, interleukin-17 receptor E, and receptor-type tyrosine-protein phosphatase C (CD45) are shown in Supplementary Figure S3. MS/MS analysis of the other discriminant *m*/*z* ions did not produce significant sequence matches (MASCOT score < 30) due to their low intensities.

**Table 2:** *In situ* protein identification. Description for database research and results from MS/MS experiments are indicated.

Mass observed	Mr (exp)	Mr (calc)	Mass error (ppm)	Mascot score	Peptide sequence/modification	Protein name	UniProt accession number	AUROC^a^	Wilcoxon test
961.49*	960.48	960.42	59	37	DGSPGGKGDR/N-Term: Methyl:2H(2) (N-term)	Collagen alpha-1(III) chain	P02461	0.26	0.001
1121.54	1120.53	1120.54	−14.7	72	R.SYVCHARSAK.G	IL-1 receptor accessory protein precursor	Q9NPH3	0.74	0.001
1137.74	1136.73	1136.53	176	98	K.NSSEGNKHHK.S	Receptor-type tyrosine-protein phosphatase C (CD45)	P08575	0.29	0.001
1138.5	1137.49	1137.58	−91.3	67	K.GEPGPMGIPGVK.G	Collagen alpha-2(IX) chain	Q14055	0.78	0.001
1140.65	1139.64	1139.51	117	67	R.DNTNEIYSGK.F	Integrin beta-1 (CD29)	P05556	0.78	0.001
1141.6	1140.59	1140.57	19.1	74	K.QEPVQKCPGR.R	Proteoglycan 4 (Lubricin)	Q92954	0.70	0.001
1510.72	1509.71	1509.65	38.8	76	R.YVDILPYDYNR./V + Phospho (Y)	Receptor-type tyrosine-protein phosphatase C (CD45)	P08575	0.28	0.001
1512.73	1511.72	1511.73	−6.67	102	R.TQHIGNQEENKSK.N	Receptor-type tyrosine-protein phosphatase C (CD45)	P08575	0.28	0.001
1671.8	1670.79	1670.80	−7.51	103	R.HGGPEFSFDLLPEAR.A	Interleukin-17 receptor E	Q8NFR9	0.71	0.001
1672.8	1671.79	1671.89	−59.2	107	R.GLPGERGRPGAPGPAGAR.G	Collagen alpha-1(I) chain precursor	P02452	0.72	0.001
1759.9*	1758.89	1758.81	42.9	96	K.GEPGPAGPQGAPGPAGEEGK.R	Collagen alpha-1(II) chain	P02458	0.28	0.001
1816.94	1815.93	1815.85	41.5	38	R.GPPGPMGPPGLAGPPGESGR.E/ + 2 Oxidation (P)	Collagen alpha-1(I) chain precursor	P02452	0.71	0.001
1818.94	1817.93	1817.82	59	145	K.NIETFTCDTQNITYR.F	Receptor-type tyrosine-protein phosphatase C (CD45)	P08575	0.72	0.001
1850.1*	1849.09	1848.89	106	68	K.TEFLSFMNTELAAFTK.N	Protein S100-A11 (Calgizzarin)	P31949	0.23	0.001
2164.1	2163.09	2163.05	16.4	92	R.SDVQFAWKHLLCPDVSYR.H	Interleukin-17 receptor E	Q8NFR9	0.75	0.001
2303.17	2302.16	2302.18	−9.16	124	K.TITTTEIMNKPEETAKPKDR.A	Proteoglycan 4 (Lubricin)	Q92954	0.6	0.001

LpPRP, leukocyte-poor platelet-rich plasma.; Mr (exp), experimental relative molecular mass; Mr (calc), calculated relative molecular mass.

* Masses more expressed in >LpPRP-0 tissues.

^a^ AUROC = Area under the receiver Operating characteristics. In the AUROC, LpPRP-1 = true positive rate; LpPRP-0 = false positive rate.

## 4. Discussion

PRP has become increasingly important worldwide in orthopedics over the past two decades. Thanks to the anti-inflammatory and regenerative nature of different growth factors and cytokines, many different indications have been established for the treatment of chronic and traumatic diseases. Due to osteoarthritis and cartilage defects, PRP has been used in orthopedics to treat cartilage defects and inflammation (5, 6, 11, 12, 28).

Growth factors and cytokines are binding to specific cellular and noncellular receptors to change the cellular expression patterns and induce an anti-inflammatory and regenerative environment. The hypothesis is that changing the cellular expression patterns in joints will first lead to change the composition of the synovial fluid, and second to induce deactivation of the inflammatory process in inflamed and activated osteoarthritis, potentially improving joint viscosity. The success rate following an injection therapy ranges from 0% to 75% according to the most recent clinical studies and consists of much longer-lasting effects than steroids or HA treatment (13, 16, 29–31).

Despite the often-reported good clinical results, it has never been investigated in a human clinical study that the reported clinical effects may be based on biological effects at the cellular level. After injection of biologically active cytokines into a joint, they bind to receptors on the cell surface over a period of time ranging from minutes to hours at the latest (8, 32, 33).

Since the clinical results after arthroscopic surgery are depending on different circumstances during and around surgery, the clinical outcome of the therapy was not the interest of this investigation. However, the positive outcome of the PRP-based autologous therapy has been previously described in different clinical studies to successfully reduce the pain, improve the ROM, and improve the ADL (18, 31, 34).

Synovitis is a regular biologic reaction to disturbances in joint homeostasis. Many types of disorders are known, such as inflammatory diseases, rheumatoid arthritis, cartilage lesions, OA, and impingement caused by meniscal tears. Our clinical model is based on synovitis induced by cartilage lesions or meniscal tears to investigate the cell biology of PRP (LpPRP)-treated synovial tissue. These disorders are known to act as joint trauma, potentially inducing an early onset of OA. Since at this early stage of the disease it is not possible to predict which patients will develop OA and which will not, for more than 6 years we have been offering preoperative ACB injection therapy to reduce inflammation and potentially reduce the risk of developing early onset of OA. We do not use leucocyte-rich PRPs because they are said to have more pro-inflammatory properties than leucocyte-poor PRPs (16). The idea is to stop the early degenerative process before reaching the cellular inflammatory phase based on the conversion from monocyte to macrophage that could potentially induce the onset of OA (12, 30, 35).

Clinical studies reporting the characteristics of these biologic therapies are numerous, as are meta-analyses. They predominantly describe clinical improvement over an extended period, longer than any other injectable therapy. Unless there are many studies analyzing the different growth factors and cytokines injected and/or expressed following PRP administration, we should always be aware that we will only find the factors we are looking for. Thus, in these “cytokine cocktails” that we produce by conditioning autologous blood according to different protocols, there may be many more and different growth factors than we expect. The step to identify the constellation of changes in growth factor and cytokine in synovial fluid has been made, according to the statement above. To the best of our knowledge, no work has been done on the changes and possible influences of PRP *in vivo* on human target tissues, such as synovial tissue. The aim of this study is to fill this gap (4, 9, 15, 29, 36).

The basis of PRP therapy and its growth factors described in the literature are, first, changes in cellular expression patterns from pro-inflammation to anti-inflammation, and second, the migration and modulation of muscular skeletal stem cells (MSCs). These effects of PRP mimic and increase the body's natural healing response to mechanical or biological induced aggression to human tissue integrity. These cellular effects might probably explain the timely extended positive clinical response to PRP administration as described in the literature (4, 9, 37).

We analyzed in a prospective, observer-blinded clinical setting 289 human synovial tissue probes harvested during arthroscopic surgery 1–7 days after a single intra-articular administration of LpPRP using MALDI MSI analysis to investigate proteomic changings being induced after/by LpPRP therapy to the synovial cell layers. MALDI MSI technology can rapidly analyze and map complex content of tissues ranging from very large proteins to small molecules with high precision. The major advantage of this approach is the capability to directly correlate mass spectral data with anatomical and pathological features since the tissue section keeps its integrity and can be stained after MALDI analysis for histomorphological interpretation. The feasibility of MALDI MSI to analyze FFPE synovial tissue to identify protein biomarker in patients with rheumatoid arthritis and osteoarthritis has been demonstrated previously (38). In this study, the outer layer of the synovial tissues of LpPRP-treated and untreated patients were analyzed and compared to explore the possible molecular changes at the peptide level.

The two groups with *n* = 128 vs. *n* = 161 patients proved to be different with an accuracy of 82%–90% in all aspects of the classification analysis. This indicates that proteomic changes have been established by LpPRP therapy after only one injection.

Stem cell attraction by chemotaxis following LpPRP therapy has been reported in animal studies, but here we can show the effect in an *in vivo* human clinical setting, showing probable enhancement of musculoskeletal stem cells (MSCs) by identification of some of their receptors. Stem cells are essential for all healing processes, so they are assumably for healing or stabilization of meniscal and cartilage defects (22, 39–42). Huard and Peng claim that even CD45+ cells might be regarded as MSCs depending on their function and importance for musculoskeletal tissue regeneration and repair (43). However, further investigations are needed to clarify the role and importance of CD45+ cells for repair and regeneration in the synovial lining of the knee joint. To our knowledge and according to the literature, we were able to demonstrate for the first time that injection therapy based on autologic-conditioned biologics (ACBs) can lead to cellular changes within synovial joints.

It is well known that joint viscosity changes by chronic synovitis and in osteoarthritis leading to improper gliding with shear forces of the cartilage finally ending up in cartilage destruction (44–46). Following the results of increased proteoglycan 4 (lubricin) in LpPRP-treated joints (LpPRP-1) compared with untreated joints (LpPRP-0), we hypothesize that the gliding capacity of the joint cartilage may be improved by higher levels of lubricin.

The third direct positive effect shown in the data analysis of this study is an improved anti-inflammation due to the activation of interleukin-17 receptor E known to reducing the inflammatory process *via* blocking interleukin-17. These three direct major effects could be demonstrated in the LpPRP-treated group (47).

In addition, because an enhancement of one pro-inflammatory peptide, in this case interleukin-1 receptor accessory precursor, was found in the LpPRP-1 group, we hypothesize that cartilage repair mechanisms, like bone repair mechanisms, are dependent on anabolic and catabolic effects for matrix reconstruction, collagen integration, and MSCs transformation and integration for cartilage preservation and restoration. Further investigations are needed to analyze the nature and mechanisms of cartilage reconstruction and reformation and to understand the complexity of healing of this very explicit tissue (48, 49).

Analysis of the untreated LpPRP-0 group demonstrates indirect effects, with obvious upregulation of elevated levels of pro-inflammatory factors, and factors known to be involved in cartilage destruction. The untreated group (LpPRP-0) shows predominant expression of three *m*/*z* peptides (*m*/*z* 961.49, 1759.9, and 1850.1, indicated with an asterisk in Table 2) having an AUROC of 0.26, 0.28, and 0.23, which indicates high intensity of these peptides in the false positive rate (LpPRP-0) group. Specifically, we found enhanced levels of protein S100-A11 (calgizzarin) that has been identified as an inflammatory mediator associated with the disease activity of rheumatoid arthritis known to be an aggressive, cartilage and joint destructing pathologic agent (50). Here, we can show that elevated levels of calgizzarin seem to be induced by joint injuries such as meniscal tears or cartilage lesions. Since diseases with higher levels of calgizzarin in joints, such as rheumatoid arthritis, are known to lead to joint destruction, we conclude that higher levels of calgizzarin might also be one of the biological factors inducing the onset of OA following meniscal tears.

Another peptide identified to be enhanced without LpPRP prevention was collagen alpha-1(III) (COL3A1). This protein contributes to the fibril assembly and biomechanical functions of both articular cartilage and meniscus. In terms of different pathologies, intracellular accumulation of COL3A1 seems to lead to pathological diameter and structure of collagen fibers, which are essential in our context for cartilage stability or reconstruction, both in meniscal fibrous cartilage and in hyaline cartilage matrix stability and restoration. Further analysis of the role of collagen alpha-1(III) chains in the onset and developing of osteoarthritis is emphasized (13, 51, 52).

Finally, enhanced levels of the third peptide, collagen alpha-1(II) chains (COL2-1), could be identified in the untreated LpPRP-0 group. COL2-1 increases in response to matrix damage if the articular cartilage is injured or undergoes advanced degenerative changes, suggesting that the potential of articular chondrocytes to repair damage to the collagen framework may be considerable (53). Interestingly, COL2-1 has been found to colocalize with collagen type 3 in the same banded fibrils in the cartilage extracellular matrix, providing evidence of a potential interaction between these collagens in maintaining tissue integrity in pathological conditions (54). Lambert et al. found in a rat model that COL2-1 peptide significantly increased the pro-inflammatory interleukin-8 (IL-8) gene expression and IL-8 production in synovial cells. Also, matrix metalloproteinase-3 (MMP-3), known to be an indicator for matrix breakdown of cartilage, was reportedly enhanced. Thus, COL2-1 triggered arthritis in a rat synovitis model induced loss of cartilage proteoglycans, cartilage structure, and subchondral bone remodeling (55, 56). These studies reinforce our finding of increased Col2-1 expression in patients not treated with LpPRP, as they may be more prone to develop OA than patients not treated with LpPRP. However, more comprehensive randomized and controlled studies confirming our findings are needed.

## 5. Conclusion

This study presented two main findings. First, by principle of serendipity, we found that in LpPRP-untreated joints, in synovitis due to meniscal tears or focal cartilage lesions, destructive peptides such as calgizzarin, COL3A1, and COL2-1, known to induce cartilage destruction and osteoarthritis, are enhanced. Second, we can show that injection therapy based on LpPRP not only downregulates these destructive proteins but also leads to cellular expression of potentially restoring therapeutically active proteins, improving gliding capacities through increased lubricin production, as well as inducing the immigration of potential cartilage-restoring MSCs. Thus, it is emphasized that a therapy based on LpPRP might be a reasonable approach to antagonize the early onset of cartilage breakdown induced by meniscal lesions.

Further analysis is emphasized to clarify and deepen these findings, and to follow up their importance for blocking the early onset of osteoarthritis by inducing this biological anti-inflammatory and possibly regenerative effects in human joints.

## Data Availability

The original contributions presented in the study are included in the article/Supplementary Material, further inquiries can be directed to the corresponding author.
