# Supplementary material for: Biological injection therapy with leukocyte-poor platelet-rich plasma induces cellular alterations, enhancement of lubricin, and inflammatory downregulation in vivo in human knees: A controlled, prospective human clinical trial based on mass spectrometry imaging analysis
Source: Front Surg. 2023 Apr 21;10:1169112. doi: 10.3389/fsurg.2023.1169112 (PMC10160617; doi:10.3389/fsurg.2023.1169112)
Supplement: Supplementary file 1 [file Datasheet1.pdf]

Supplementary Tables

Table S1. Patient characteristics

N. Patient	LpPRP Treatment *	Age	Gender §
1	0	82	F
2	0	80	F
3	0	67	M
4	1	73	M
5	0	69	M
6	0	69	M
7	0	73	F
8	0	72	M
9	0	69	F
10	0	56	F
11	0	56	F
12	0	54	M
13	1	72	M
14	0	71	F
15	0	60	F
16	0	61	M
17	0	71	M
18	0	84	F
19	0	35	F
20	0	83	F
21	1	44	F
22	1	59	M
23	0	36	F
24	0	47	M
25	0	47	M
26	0	53	F
27	0	65	M
28	0	67	F
29	0	57	F
30	0	41	F
31	0	72	F
32	0	61	F
33	0	71	F
34	1	52	M
35	1	78	F
36	1	62	F
37	1	67	F
38	1	50	M
39	1	53	M
40	1	75	F
41	1	66	M
42	1	62	M
43	1	64	F
44	1	67	F
45	1	57	M
46	1	63	M
47	1	59	F
48	1	58	M
49	1	48	F
50	1	74	F
51	1	51	M
52	1	68	F
53	1	51	F
54	1	70	F
55	1	33	M
56	1	65	M
57	1	65	F
58	1	49	F
59	0	59	U

60	0	67	F
61	0	66	F
62	0	62	M
63	0	78	M
64	0	69	F
65	0	57	M
66	0	63	F
67	0	71	M
68	0	67	M
69	0	90	F
70	0	60	M
71	0	72	F
72	0	76	F
73	0	52	F
74	0	48	M
75	0	69	F
76	0	79	F
77	0	82	F
78	0	75	F
79	0	62	F
80	0	62	M
81	0	47	M
82	0	61	F
83	0	66	F
84	0	58	M
85	0	72	F
86	0	64	M
87	0	55	M
88	1	64	M
89	1	69	F
90	1	60	F
91	1	50	M
92	1	70	F
93	1	77	M
94	1	72	F
95	1	76	F
96	1	52	F
97	1	69	F
98	1	59	M
99	1	64	M
100	1	31	M
101	1	63	M
102	1	30	M
103	1	58	M
104	1	64	F
105	1	60	F
106	1	47	M
107	1	55	F
108	1	70	M
109	1	72	M
110	1	41	M
111	1	63	F
112	1	58	M
113	1	58	M
114	1	65	M
115	1	57	M
116	1	58	M
117	0	52	M
118	0	61	F
119	0	70	U
120	0	30	M
121	0	63	M
122	0	53	M

123	0	63	F
124	0	54	F
125	0	71	F
126	0	65	F
127	0	73	M
128	0	87	F
129	0	83	F
130	0	59	M
131	0	68	F
132	0	21	F
133	0	57	F
134	0	47	F
135	0	53	M
136	0	77	F
137	0	64	F
138	0	82	F
139	0	45	M
140	0	59	M
141	0	55	F
142	0	75	M
143	0	67	F
144	0	60	F
145	1	56	M
146	1	62	F
147	1	40	M
148	1	67	F
149	1	44	M
150	1	73	F
151	1	50	F
152	1	65	F
153	1	46	F
154	1	70	F
155	1	64	F
156	1	53	F
157	1	69	M
158	1	60	M
159	1	72	F
160	1	53	F
161	1	68	F
162	1	60	F
163	1	44	M
164	1	66	F
165	1	61	F
166	1	67	F
167	1	61	F
168	1	82	M
169	1	69	M
170	1	72	F
171	1	62	F
172	1	50	M
173	1	78	F
174	0	69	M
175	0	62	M
176	0	34	M
177	0	67	M
178	0	58	M
179	0	55	F
180	0	71	M
181	0	65	M
182	0	58	M
183	0	61	F
184	0	73	F
185	0	37	M

186	0	61	M
187	0	71	F
188	0	61	F
189	0	64	F
190	0	81	F
191	0	70	M
192	0	70	M
193	0	83	F
194	0	63	F
195	0	52	M
196	0	45	F
197	0	64	F
198	0	54	M
199	0	61	F
200	0	67	M
201	0	53	M
202	0	62	F
203	0	89	F
204	1	33	M
205	1	74	M
206	1	81	F
207	1	67	F
208	1	63	M
209	1	67	F
210	1	69	F
211	1	64	F
212	1	64	M
213	1	31	M
214	1	77	F
215	1	53	M
216	1	63	M
217	1	60	M
218	1	80	F
219	1	42	M
220	1	61	F
221	1	47	F
222	1	57	M
223	1	53	F
224	1	65	F
225	1	31	M
226	1	54	F
227	1	86	F
228	1	70	F
229	1	64	M
230	1	72	F
231	1	55	F
232	1	45	F
233	0	75	M
234	0	75	U
235	0	70	M
236	0	70	F
237	0	88	F
238	0	72	M
239	0	54	M
240	0	54	F
241	0	25	M
242	0	54	F
243	0	79	M
244	0	68	M
245	0	24	M
246	0	42	F
247	0	76	F
248	0	46	F

249	0	73	F
250	0	66	F
251	0	59	M
252	0	59	F
253	0	51	F
254	0	53	M
255	0	52	F
256	0	64	M
257	0	67	M
258	0	78	F
259	0	61	M
260	0	58	F
261	1	47	F
262	1	38	M
263	1	55	F
264	1	60	F
265	1	61	F
266	1	59	F
267	1	69	M
268	1	36	M
269	1	57	F
270	1	59	M
271	1	47	M
272	1	47	M
273	1	60	M
274	1	59	F
275	1	75	F
276	1	35	M
277	1	57	M
278	1	50	M
279	1	64	M
280	1	48	M
281	1	52	M
282	1	60	F
283	1	52	M
284	1	42	F
285	1	55	M
286	1	51	F
287	1	51	M
288	1	44	M
289	1	68	F
290	0	74	F
291	0	62	M
292	0	64	M
293	0	54	M
294	0	85	M
295	0	63	M
296	0	64	F
297	0	77	M
298	0	66	M
299	0	65	F
300	0	73	F
301	0	55	M
302	0	69	M
303	0	84	F
304	0	78	M
305	0	52	M
306	0	70	M
307	0	69	M
308	0	66	F
309	0	40	M
310	0	49	M
311	0	76	F

312	0	66	F
313	0	50	F
314	0	55	M
315	0	76	M
316	0	67	M
317	0	73	F
318	0	70	F
319	0	58	M
320	0	62	M
321	0	60	F
322	0	68	F
323	0	59	F
324	0	71	F
325	0	69	M
326	0	58	M
327	0	57	M
328	0	56	F
329	0	65	M
330	0	60	M
331	1	39	M
332	1	83	M
333	1	68	F
334	1	68	F
335	1	69	M
336	1	50	M
337	1	57	M
338	1	80	F
339	1	83	F
340	1	57	M
341	1	42	M
342	1	55	M
343	1	58	F
344	1	70	F
345	1	48	F
346	1	39	M
347	1	47	F
348	1	72	F
349	0	41	F
350	0	52	M
351	0	80	F
352	0	63	M
353	0	92	F
354	0	57	M
355	0	82	F
356	0	26	M
357	0	56	M
358	0	51	F
359	0	57	M
360	0	73	F
361	0	68	F
362	0	58	M
363	0	81	F
364	0	61	M
365	0	40	F
366	0	63	F
367	0	54	M

[*] LpPRP= 0: patients non-treated with LpPRP; LpPRP= 1: patients treated with LpPRP

[§] F= Female; M= Male; U= Unknown

Table S2. Trypsin and matrix deposition methods for TM-Sprayer device.

Method deposition	Reagent concentration (mg/mL)	Flow rate (mL/min)	Velocity of the nozzle (mm/min)	Track spacing (mm)	Temperature of the nozzle (C°)	Number of layers	Pressure (psi)
Trypsin	0.03	0.015	750	2	30	16	10
Matrix	10	0.12	1200	3	75	4	10

Table S3. Univariate statistical Wilcoxon test to compare spectral data from TMAs random groups and from muscle tissues random groups

		TMA group 1	Muscle group 1	TMA group 2	Muscle group 2
	LpPRP-0 - LPPRP-1	TMA 2,3,4 vs TMA 5,6,7	Muscle (TMA 2,3,4) vs Muscle (TMA 5,6,7)	TMA 2,4,6 vs TMA 3,5,7	Muscle (TMA 2,5,7) vs Muscle (TMA 3,4,6)
m/z	Wilcoxon Rank Sum Test p-value	Wilcoxon Rank Sum Test p-value	Wilcoxon Rank Sum Test p-value	Wilcoxon Rank Sum Test p-value	Wilcoxon Rank Sum Test p-value
602.28	0	0.36	1.00	0.82	0.39
603	0	0.36	1.00	0.82	0.39
604.32	0	0.36	0.84	0.82	0.39
605.3	0	0.36	1.00	0.82	0.39
606.12	0	0.36	0.67	0.82	1.00
606.32	0	0.36	0.67	0.82	1.00
617.33	0	0.36	1.00	0.82	0.39
618.3	0	0.36	0.84	0.82	0.39
619.28	0	0.36	0.84	0.82	0.39
620.3	0	0.36	1.00	0.82	0.39
621.328	0	0.36	0.84	0.82	0.39
624.3	0	0.36	0.84	0.82	0.39
628.29	0	0.36	0.67	0.82	0.93
629.318	0	0.36	0.67	0.82	0.93
630.341	0	0.36	1.00	0.82	0.39
631.364	0	0.36	0.68	0.82	0.88
632.338	0	0.36	1.00	0.82	0.39
633.313	0	0.36	1.00	0.82	0.39
640.279	0	0.36	0.84	0.82	0.39
644.03	0	0.36	0.67	0.82	1.00
644.323	0	0.36	0.68	0.82	0.93
645.053	0	0.36	0.67	0.82	1.00
645.346	0	0.36	0.68	0.82	0.93
646.076	0	0.36	0.67	0.82	1.00
650.071	0	0.36	0.68	0.82	0.39
651.094	0	0.36	0.84	0.82	0.39
661.374	0	0.36	0.84	0.82	0.65
664.345	0	0.36	0.84	0.82	0.39
666.05	5.11E-14	0.52	1.00	0.82	1.00
666.343	0	0.36	0.84	0.52	0.93
667.073	0	0.36	1.00	0.82	1.00
668.048	0	0.36	0.68	0.82	0.88
669.314	0	0.36	0.84	0.82	0.39
671.361	0	0.36	0.68	0.82	0.39
672.384	0	0.36	1.00	0.81	0.39
678.327	0	0.36	1.00	0.81	0.65
679.301	0	0.36	1.00	0.82	0.93
682.03	0	0.52	0.68	0.82	0.39
682.273	0	0.36	1.00	0.82	0.39
688.363	0	0.36	1.00	0.82	0.39
694.355	0	0.36	1.00	0.82	0.39
700.347	0	0.36	1.00	0.82	0.88

701.37	0	0.36	1.00	0.82	0.88
702.345	0	0.36	0.68	0.82	0.88
703.368	0	0.36	0.68	0.82	0.39
704.342	0	0.36	0.68	0.82	0.39
715.401	0	0.36	0.84	0.82	0.39
717.398	0	0.36	0.84	0.82	0.65
718.372	0	0.36	1.00	0.82	0.39
730.357	0	0.36	0.68	0.82	0.88
731.331	0	0.36	1.00	0.82	0.39
732.354	0	0.36	0.68	0.82	0.39
733.377	0	0.36	1.00	0.82	0.65
738.346	0	0.36	0.68	0.82	0.65
740.344	0	0.36	1.00	0.82	0.39
758.369	0	0.36	0.84	0.82	0.39
759.392	0	0.36	1.00	0.82	0.39
760.366	0	0.36	1.00	0.82	0.65
768.307	0	0.36	0.84	0.82	0.39
771.377	0	0.36	1.00	0.82	0.65
772.4	0	0.36	1.00	0.82	0.39
775.371	0	0.36	0.84	0.82	0.39
776.346	0	0.36	1.00	0.82	0.88
777.369	0	0.36	1.00	0.82	0.39
785.407	0	0.36	0.84	0.82	0.39
788.427	0	0.36	1.00	0.81	0.93
789.451	0	0.36	1.00	0.82	0.88
795.394	0	0.36	0.68	0.82	0.39
796.417	0	0.36	0.84	0.82	0.39
797.391	0	0.36	1.00	0.82	0.39
815.417	0	0.36	0.68	0.82	0.39
816.44	0	0.36	0.68	0.82	0.39
817.414	0	0.36	1.00	0.82	1.00
828.424	0	0.52	0.68	0.82	0.93
831.445	0	0.36	1.00	0.82	1.00
833.101	0	0.36	0.67	0.82	1.00
833.344	0	0.36	0.67	1.00	0.39
834.075	0	0.36	0.67	0.82	0.39
834.368	0	0.36	0.67	0.82	0.88
836.414	0.0072	1.00	0.68	0.52	1.00
837.437	0	0.36	1.00	0.82	0.88
838.411	0	0.36	1.00	0.82	1.00
839.093	0	0.36	0.67	0.82	0.93
840.457	0	0.36	1.00	0.82	0.65
841.48	0	0.36	0.68	0.82	0.39
842.503	0	0.52	0.84	0.82	0.39
843.526	0	0.36	0.84	0.82	0.93
844.501	0	0.36	1.00	0.82	1.00
845.378	0	0.36	0.84	0.82	1.00

848.447	0	0.36	1.00	0.82	0.93
852.442	0	0.52	0.84	0.82	1.00
853.416	0	0.52	0.84	0.82	0.39
854.439	0	0.36	1.00	0.52	0.65
855.072	0	0.36	0.67	0.82	0.88
856.095	0	0.36	0.67	0.52	0.88
856.485	0	0.36	0.84	0.82	0.65
857.07	0	0.36	0.67	0.82	1.00
857.508	0	0.36	0.68	0.82	1.00
858.434	0	0.36	0.84	0.81	1.00
861.113	0	0.36	1.00	0.82	1.00
862.088	2.56E-13	0.36	1.00	0.82	0.88
864.475	0	0.36	1.00	0.82	0.39
868.469	0	0.36	0.84	0.82	0.39
869.444	0	0.36	0.84	0.82	0.65
870.516	0	0.36	0.84	0.82	0.39
871.051	0	0.52	0.84	0.82	0.39
871.539	0	0.36	0.68	0.82	0.39
872.074	0	0.36	0.84	0.82	1.00
872.416	0	0.36	0.84	0.82	0.65
873.39	0	0.36	0.84	0.82	0.39
874.413	0	0.36	1.00	0.82	0.39
875.387	0	0.36	1.00	0.82	0.39
877.092	8.19E-08	0.73	0.84	0.82	0.39
878.067	1.07E-09	0.73	0.84	0.82	0.88
880.454	0	0.73	0.84	0.82	0.93
886.446	0	0.36	0.84	0.82	0.88
887.42	0	0.36	1.00	0.82	0.93
890.392	0	0.36	1.00	0.82	0.93
891.415	0	0.36	1.00	0.82	0.39
893.023	0	0.73	0.68	0.82	0.39
894.436	0	0.36	0.68	0.82	0.93
896.384	0	0.36	1.00	0.82	0.39
898.479	0	0.73	0.84	0.82	0.88
899.502	0	0.52	0.68	0.82	0.88
900.476	0	0.36	1.00	0.82	0.39
901.5	0	0.36	1.00	0.82	0.93
902.474	0	0.36	1.00	0.82	0.88
905.446	0	0.52	0.84	0.82	0.39
906.42	0	0.36	1.00	0.82	0.39
908.417	0	0.36	0.68	0.82	0.88
910.415	0	0.36	0.68	0.82	0.88
912.412	0	0.36	0.68	0.82	0.65
913.435	0	0.52	0.68	0.82	0.88
914.458	0	0.36	0.68	0.82	0.88
915.433	0	0.36	0.68	0.82	0.88
916.456	0	0.36	1.00	0.82	0.93

920.45	0	0.36	1.00	0.82	0.93
936.43	0	0.36	0.68	0.82	0.88
940.473	0	0.36	1.00	0.82	0.88
944.468	5.51E-06	0.36	0.84	0.82	0.93
945.442	0	0.36	0.68	0.82	0.88
946.417	0	0.36	0.68	0.82	0.88
957.524	0	0.36	1.00	0.82	0.88
958.498	0	0.36	0.68	0.82	0.88
960.447	0	0.36	0.68	0.82	0.65
961.421	0	0.36	1.00	0.82	0.65
962.444	0	0.36	1.00	0.82	0.39
964.442	0	0.36	0.68	0.82	0.39
971.506	0	0.36	0.68	0.82	0.65
972.48	0	0.36	0.84	0.82	0.65
974.429	0	0.36	0.68	0.82	0.65
975.452	0	0.36	0.68	0.82	1.00
976.475	0	0.36	0.68	0.82	1.00
977.449	0	0.36	0.68	0.82	0.93
978.472	0	0.36	0.68	0.82	0.88
979.495	0	0.36	0.68	0.82	1.00
980.47	0.006	0.52	0.68	1.00	1.00
990.408	0	0.36	0.68	0.81	0.93
998.446	0	0.36	0.68	0.81	0.88
999.469	0	0.36	0.68	0.82	0.93
1000.492	0	0.36	0.68	0.82	0.93
1002.441	0	0.36	0.68	0.82	0.93
1004.487	0	0.36	0.68	0.82	0.93
1014.474	0	0.36	1.00	0.82	0.93
1015.497	0	0.36	1.00	0.82	0.65
1028.505	0	0.36	1.00	0.82	1.00
1029.528	0	0.36	1.00	0.82	0.88
1032.548	0	0.52	0.68	0.82	0.93
1033.571	0	0.36	1.00	0.82	0.65
1034.546	4.39E-06	0.36	0.84	0.82	0.93
1036.494	0	0.36	1.00	0.82	0.93
1037.517	0	0.36	1.00	0.82	0.93
1045.556	0	0.36	0.84	0.52	0.93
1046.579	0	0.36	0.84	0.52	1.00
1047.553	0	0.36	0.68	0.52	1.00
1052.473	0	0.36	1.00	0.82	0.88
1053.497	0	0.36	0.68	0.82	0.93
1060.073	0	0.36	0.67	0.52	0.93
1061.096	0	0.73	0.67	0.52	0.93
1066.114	0	0.36	0.67	0.82	0.93
1067.089	0	0.36	0.67	0.82	1.00
1068.501	0	0.36	0.84	0.82	1.00
1071.522	0	0.36	0.68	0.82	0.88

1082.093	0	0.73	0.84	0.82	0.93
1083.068	0	0.73	0.84	0.52	0.93
1087.55	0	0.52	0.68	0.52	1.00
1088.524	0	0.36	0.68	0.82	1.00
1089.547	0	0.36	0.84	0.82	0.93
1093.542	0	0.36	0.68	0.82	0.93
1094.565	0	0.52	0.68	0.82	0.93
1095.588	0	0.52	0.67	0.82	0.93
1096.562	0	0.52	0.67	0.81	0.93
1097.585	0	0.52	0.67	1.00	1.00
1106.598	6.29E-07	0.73	0.68	0.82	0.93
1107.572	0	0.73	0.84	0.82	0.93
1110.544	0	0.36	0.84	0.82	0.93
1111.616	0	0.52	0.67	0.82	0.93
1112.59	0	0.52	0.67	0.82	1.00
1113.613	0	0.36	0.68	0.82	0.93
1116.536	0	0.36	1.00	0.82	0.65
1117.511	0	0.36	0.84	0.82	0.93
1118.534	0	0.36	0.84	0.82	1.00
1127.546	2.49E-10	1.00	0.84	0.82	1.00
1128.569	0	0.52	0.68	0.52	1.00
1130.567	0	0.36	0.68	0.82	1.00
1131.541	0	0.36	0.68	0.82	0.93
1132.564	0	0.36	0.68	0.82	0.39
1133.538	0	0.52	0.67	0.82	0.39
1134.562	0	0.36	0.68	0.82	0.39
1138.556	0	0.36	1.00	0.82	0.39
1139.579	0	0.36	1.00	0.82	0.65
1140.554	0	0.52	0.84	0.82	0.39
1141.577	0	0.36	1.00	0.82	0.39
1144.549	0	1.00	0.68	0.82	0.39
1146.595	0	0.36	0.68	0.82	0.93
1149.566	0	0.52	0.68	0.82	0.39
1150.541	0	0.52	0.68	0.82	0.88
1154.584	0	0.52	0.68	0.82	0.88
1155.559	0	0.36	0.68	0.82	0.93
1156.533	0	0.36	0.68	0.82	0.88
1157.507	0	0.36	0.68	0.82	0.88
1160.528	0	0.52	0.68	0.82	0.88
1161.551	0	0.36	0.68	0.82	1.00
1165.546	0.000201782	0.73	1.00	1.00	0.88
1170.563	0	0.36	0.68	1.00	1.00
1171.538	0	0.52	0.68	0.81	0.88
1173.535	0	0.36	1.00	1.00	0.65
1174.509	0	0.36	0.84	0.52	1.00
1176.507	0	0.36	0.68	0.81	0.93
1177.53	0	0.36	0.67	1.00	0.39

1184.496	0	0.36	0.67	1.00	0.93
1192.535	0	0.36	0.68	1.00	0.93
1198.673	0	0.36	0.84	0.82	0.93
1199.696	0	0.36	0.84	0.81	0.93
1212.606	0	0.36	0.68	0.52	1.00
1214.604	0	0.52	0.68	0.81	1.00
1229.56	2.71E-10	0.36	0.68	0.52	0.93
1230.583	0	0.52	0.68	0.52	1.00
1235.601	0	0.73	0.68	0.52	0.93
1236.624	0	0.52	0.84	0.52	1.00
1237.598	0	0.52	0.84	0.52	0.88
1240.667	0.027006868	1.00	0.68	0.52	1.00
1242.616	0	0.52	0.68	0.52	1.00
1243.639	0.014865884	0.52	0.68	0.52	0.93
1244.613	0	0.52	0.68	0.52	0.88
1267.656	0	0.52	0.68	0.81	0.93
1268.679	0	0.52	0.68	0.52	1.00
1269.654	0	0.36	0.84	0.81	1.00
1271.067	0	0.36	0.67	0.81	1.00
1287.63	0	0.36	1.00	0.82	1.00
1289.628	0	0.52	0.68	0.52	0.93
1297.617	0	0.52	0.68	0.52	0.88
1298.592	0	0.36	0.68	0.81	0.93
1303.609	0	0.52	0.67	0.81	0.93
1304.584	0	0.52	0.68	0.52	1.00
1305.607	0	0.73	0.68	0.52	1.00
1314.668	0	0.52	0.84	1.00	1.00
1315.691	0	0.52	1.00	1.00	0.93
1321.635	0.000632113	0.52	0.67	1.00	1.00
1322.658	4.99E-16	0.73	0.67	0.82	1.00
1323.632	0	0.73	0.67	0.82	1.00
1324.606	0	1.00	0.68	0.52	1.00
1325.581	0	0.36	0.68	0.52	1.00
1326.604	0	0.36	0.68	0.52	0.93
1337.663	0	0.36	1.00	0.81	0.93
1338.686	0	0.36	0.68	0.81	0.93
1340.586	0	1.00	0.67	0.81	1.00
1341.56	0	1.00	0.68	1.00	1.00
1342.632	2.69E-13	0.73	0.68	1.00	1.00
1343.655	0.006748812	0.73	0.68	0.82	1.00
1359.634	3.43E-06	0.36	0.68	1.00	1.00
1366.6	3.92E-06	0.73	0.68	1.00	0.93
1367.624	5.55E-13	0.52	0.84	1.00	0.88
1368.647	1.41E-13	0.52	0.84	1.00	0.65
1401.628	0	0.36	0.68	1.00	0.88
1428.715	0	0.73	0.68	1.00	0.93
1429.689	0	0.73	0.68	1.00	0.93

1430.712	0	0.73	0.84	1.00	0.93
1459.699	3.52E-06	0.73	0.68	1.00	0.93
1460.673	0.007881892	0.73	0.68	0.52	0.93
1461.696	8.10E-08	1.00	0.84	1.00	0.39
1465.691	0.00133129	0.73	0.84	1.00	0.39
1467.688	3.92E-06	1.00	0.84	1.00	0.39
1477.724	0	0.36	0.68	1.00	0.39
1478.747	0	0.52	0.67	1.00	0.39
1479.77	0	0.36	0.67	1.00	0.39
1482.693	0.050420561	1.00	0.68	1.00	0.39
1487.662	0.002234835	0.73	0.84	1.00	0.65
1493.752	0	0.52	0.68	1.00	0.65
1494.726	0	1.00	0.68	1.00	0.65
1495.749	0	0.52	0.67	1.00	0.65
1496.772	0	0.52	0.67	1.00	0.93
1497.747	1.26E-06	0.73	0.68	1.00	0.88
1508.708	0	0.52	0.68	1.00	0.88
1509.731	0	0.52	0.68	1.00	0.65
1510.705	0	0.36	0.84	1.00	0.88
1511.728	0	0.36	1.00	1.00	0.39
1515.723	0	0.73	1.00	0.82	0.93
1529.754	0.035546399	0.52	0.68	0.82	1.00
1530.728	0	1.00	1.00	0.52	1.00
1531.702	0	0.73	1.00	1.00	1.00
1532.725	0	0.52	0.84	0.81	1.00
1533.7	0	0.73	0.68	1.00	1.00
1546.805	0	0.73	1.00	1.00	1.00
1547.779	0	1.00	1.00	0.82	1.00
1548.802	0	1.00	0.68	0.52	1.00
1561.809	0	1.00	0.67	1.00	1.00
1562.784	0	0.73	0.67	1.00	1.00
1563.807	0	0.73	0.68	1.00	1.00
1564.781	0	0.73	0.68	1.00	0.93
1568.776	0	0.73	1.00	0.82	0.88
1573.696	0	1.00	0.84	0.82	1.00
1584.755	0	1.00	0.68	1.00	0.88
1585.778	0	0.73	0.68	1.00	0.88
1586.801	0	0.73	0.84	1.00	1.00
1587.776	0	0.73	0.84	0.52	0.93
1619.783	0	0.73	0.68	1.00	1.00
1626.749	0	0.52	0.68	0.82	0.93
1627.772	0	0.52	0.84	1.00	0.93
1628.747	0	0.52	0.84	0.82	1.00
1669.815	0	0.73	0.68	0.52	1.00
1670.838	0	1.00	0.68	0.82	1.00
1671.812	0	0.73	0.68	0.82	1.00
1685.794	0	0.73	0.68	0.82	0.93

1686.817	0	0.36	0.68	1.00	0.93
1690.861	0	1.00	0.68	0.52	1.00
1691.835	0	1.00	0.68	1.00	0.93
1692.858	0	1.00	0.68	0.81	0.93
1706.791	0	0.73	0.68	0.81	0.65
1707.814	0	0.73	0.68	0.81	0.88
1708.789	0	1.00	0.68	1.00	1.00
1710.883	0	0.73	1.00	1.00	1.00
1711.858	0	0.73	0.84	1.00	0.93
1742.793	0	0.73	0.68	1.00	1.00
1743.767	0	1.00	0.84	1.00	1.00
1744.79	0	1.00	0.84	1.00	1.00
1753.852	0.052456291	1.00	0.68	0.82	0.65
1756.823	0	0.36	0.68	0.82	0.39
1757.798	0	0.52	0.68	0.82	0.65
1766.859	0	1.00	0.68	1.00	0.88
1767.833	0	1.00	0.68	1.00	0.93
1775.921	0	1.00	0.84	1.00	0.88
1776.895	0	0.73	0.84	1.00	0.88
1790.877	0	1.00	1.00	1.00	0.93
1791.9	0	0.73	1.00	1.00	0.39
1816.891	0	1.00	0.68	1.00	0.39
1817.915	0	1.00	0.68	0.82	0.93
1818.889	0	1.00	0.68	0.82	0.93
1832.871	0	0.52	0.68	0.82	1.00
1833.942	2.50E-16	0.73	0.84	0.81	0.93
1834.917	0	0.73	0.68	1.00	1.00
1835.94	0	0.73	0.84	0.82	1.00
1836.914	0	0.36	0.68	0.82	0.93
1850.896	0	0.73	0.68	0.81	0.93
1851.87	0	0.73	0.68	0.82	0.65
1852.893	0	0.73	0.84	0.82	0.93
1922.948	3.51E-14	0.52	0.68	0.81	1.00
1923.923	0	0.73	0.68	0.52	1.00
1924.946	0	0.73	0.68	0.52	1.00
1961.971	0	0.73	0.68	0.52	1.00
1962.994	0	0.52	0.68	0.52	1.00
1963.968	0	0.52	0.68	0.81	1.00
2056.092	0	0.52	0.84	0.52	0.93
2057.017	0	0.73	0.68	0.52	0.93
2057.992	0	0.52	0.68	0.52	1.00
2059.015	0	0.52	1.00	0.81	1.00
2072.071	0	0.52	0.67	0.82	0.93
2073.094	0	0.73	0.67	0.81	0.93
2074.068	0	0.52	0.67	1.00	1.00
2089.073	0	0.73	0.67	1.00	1.00
2090.048	0	0.52	0.67	1.00	1.00

2104.127	0	1.00	0.68	0.52	0.39
2105.101	0	0.73	0.68	0.52	0.39
2106.075	0	0.73	0.67	0.52	0.39
2107.098	0	0.73	0.68	0.52	1.00
2115.137	0	0.52	0.67	1.00	1.00
2116.16	0	0.52	0.67	0.52	0.93
2117.134	0	0.52	0.68	0.82	0.93
2118.157	0	0.52	0.68	0.52	0.88
2138.131	0	0.73	0.67	0.52	1.00
2154.11	0	0.73	0.68	0.82	0.93
2164.049	0	1.00	0.68	0.52	1.00
2165.072	0	0.52	0.67	0.82	0.93
2200.05	0	0.73	0.84	0.52	0.93
2216.127	0	0.52	0.68	0.82	0.93
2217.101	0	0.73	1.00	0.52	0.93
2218.124	0	0.73	0.84	1.00	0.93
2219.099	0	0.73	0.68	1.00	1.00
2454.256	0	0.52	0.68	0.52	1.00
2455.279	0	0.52	0.68	0.82	1.00
2456.253	0	0.52	0.68	1.00	1.00
2540.192	0	0.52	0.84	0.81	0.93
2674.31	0	0.36	1.00	0.82	0.65
2675.333	0	0.36	0.84	0.82	0.65
2676.308	0	0.36	0.68	0.81	0.93
2690.338	0	0.36	0.84	0.52	1.00
2691.312	0	0.36	0.68	0.82	0.88
2692.336	0	0.36	0.84	0.52	0.93
2705.294	0	0.52	0.84	1.00	0.93
2706.317	0	0.36	0.68	0.52	1.00
2707.292	0	0.36	0.68	1.00	0.88
2708.315	0	0.36	0.68	0.82	0.93
2727.363	0	0.52	0.68	0.82	0.93
2728.337	0	0.52	0.68	0.82	1.00
2729.36	0	0.52	0.84	0.82	1.00
2869.422	0	0.52	0.68	0.52	0.93
2870.445	0	0.52	0.68	0.52	1.00
2871.419	0	0.52	0.68	0.82	0.93
2872.442	0	0.52	0.84	1.00	0.88
2950.487	0	0.52	0.68	0.82	1.00
2951.51	0	0.52	0.68	0.52	1.00
2952.533	0	0.52	0.68	0.82	0.65
2953.507	0	0.52	0.68	0.52	1.00
2959.451	0	0.36	0.68	0.52	1.00
2960.425	0	0.52	0.67	0.52	0.93
2961.399	0	0.36	0.68	0.81	1.00
2962.422	0	0.52	0.68	1.00	1.00
3085.53	0	0.36	1.00	0.82	1.00

3086.5	0	0.36	1.00	0.82	0.88
3100.53	0	0.36	0.84	0.81	1.00
3101.5	0	0.36	1.00	0.82	1.00
3102.53	0	0.36	1.00	0.82	0.93
3103.5	0	0.36	1.00	0.82	0.93

Supplementary Figures

(A)

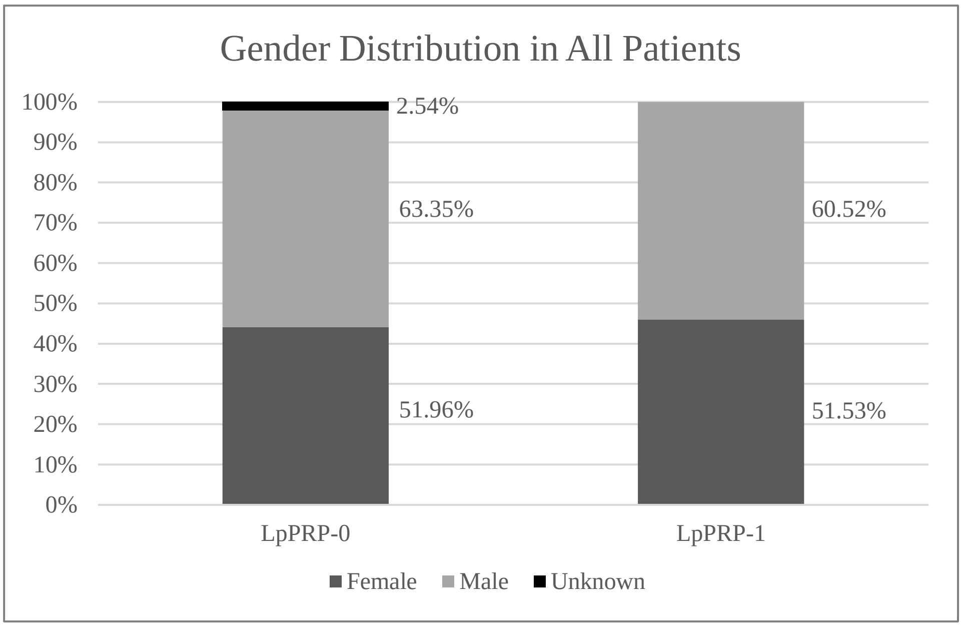

(B)

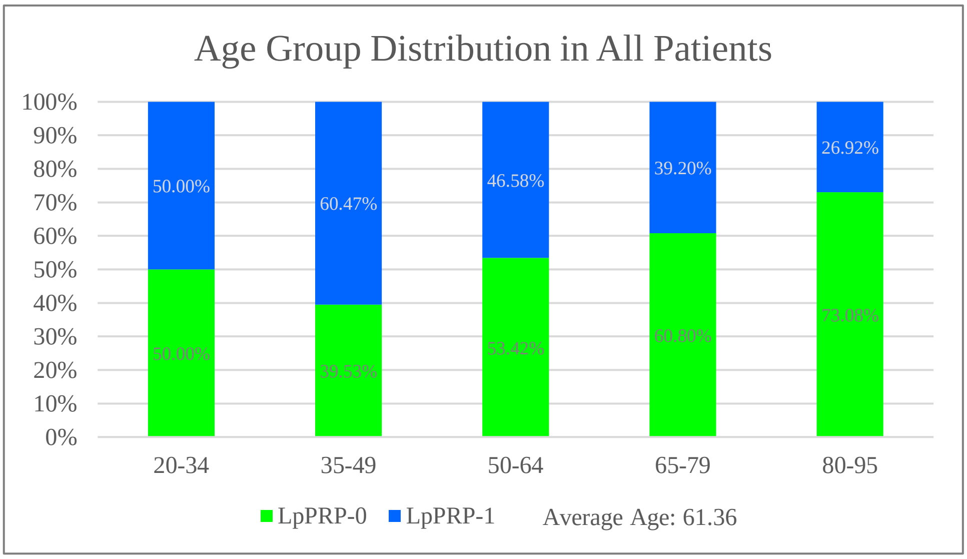

Figure S1. Patients' distribution according to gender (A) and age (B).

(A)

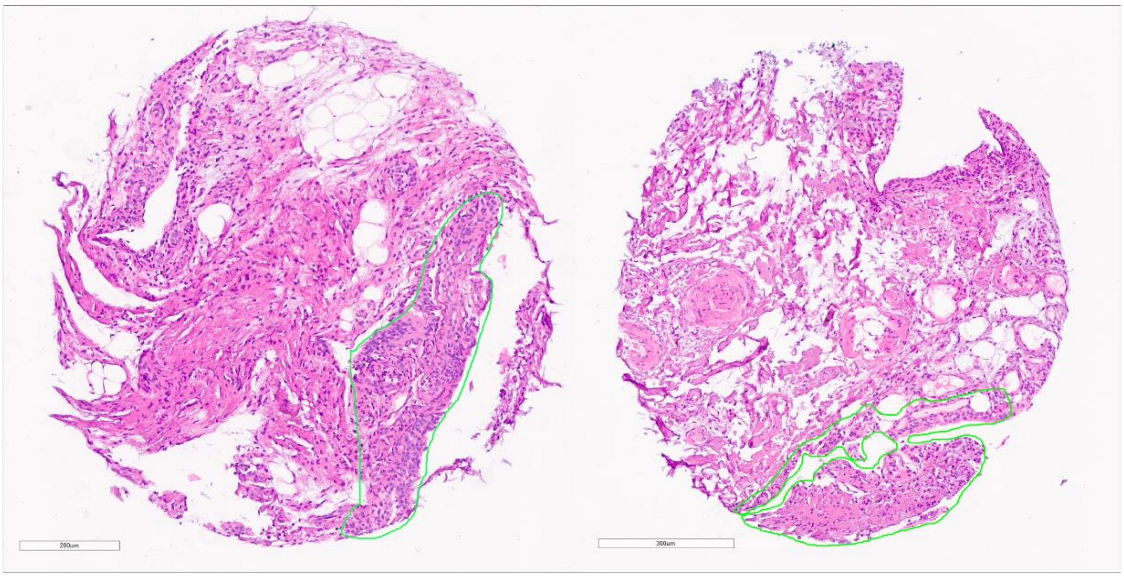

(B)

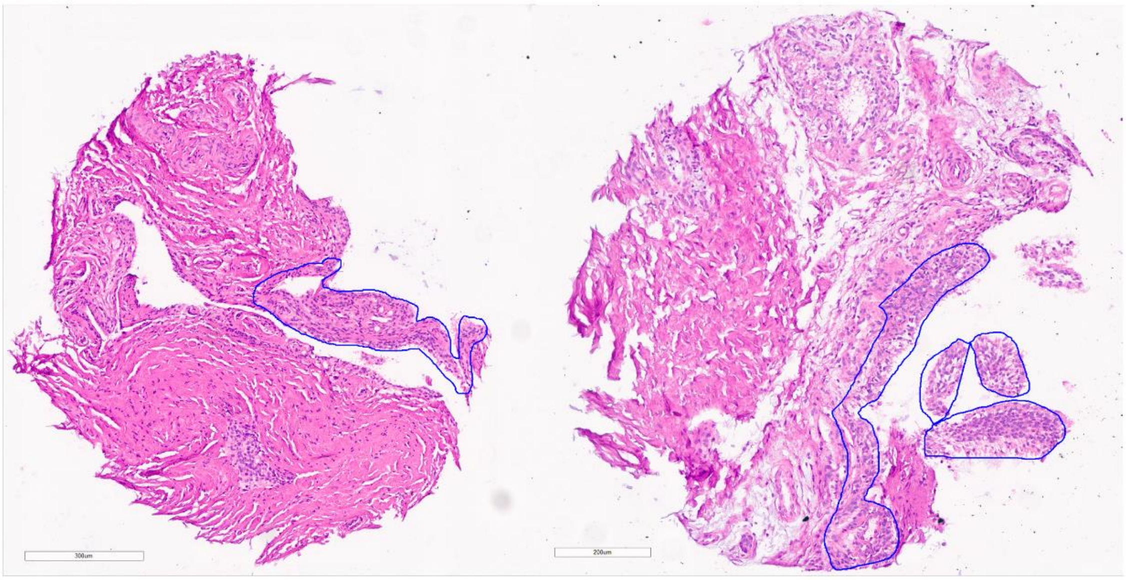

Figure S2. Hematoxylin & eosin stained sections of representative tissues from two individual patients non-treated (A) and two treated (B) with LpPRP. Annotated regions (green in LpPRP-untreated, blue in LpPRP-treated) show inflammed areas from which spectral data was extracted for classification analysis.

(A)

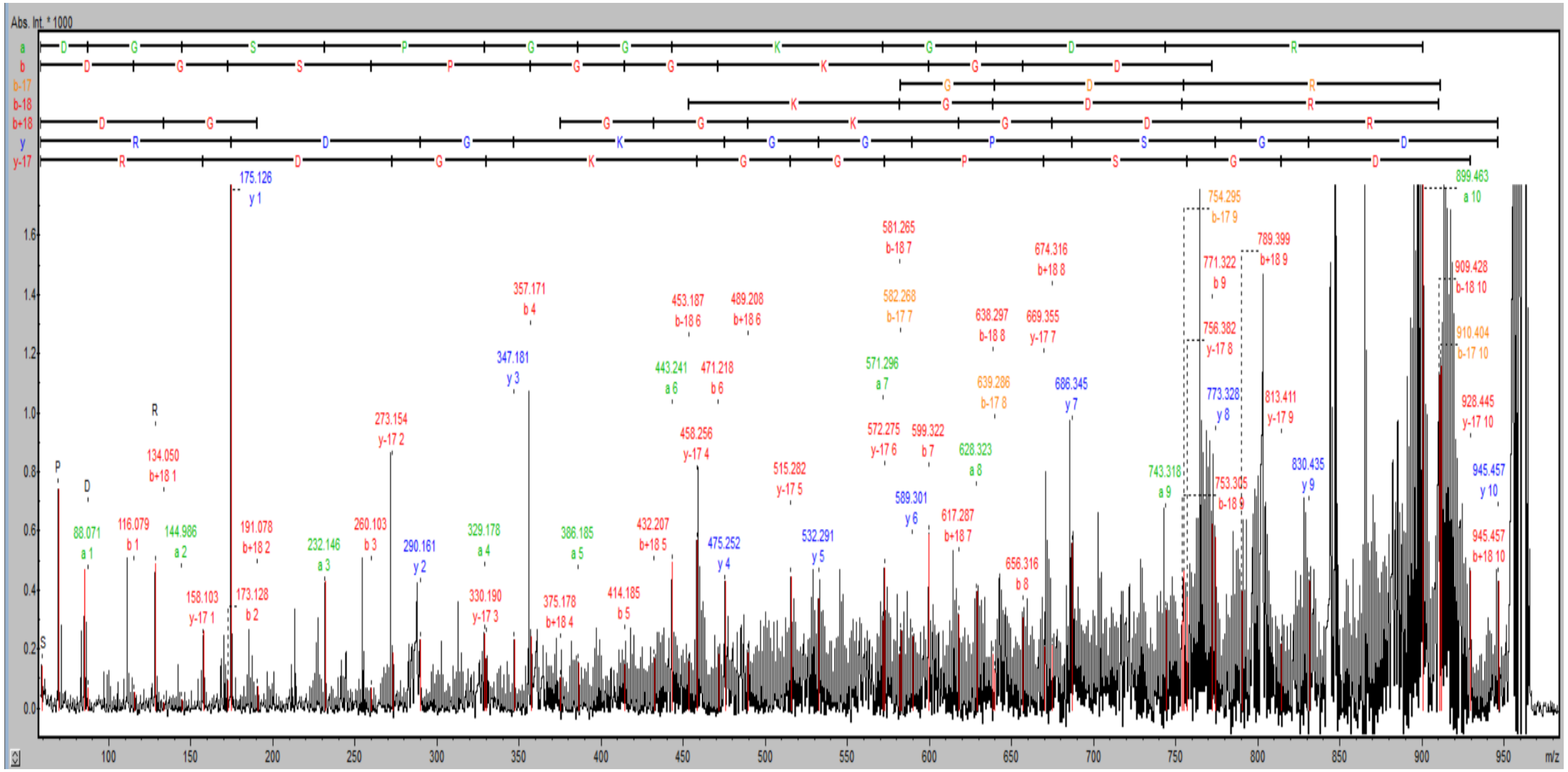

(B)

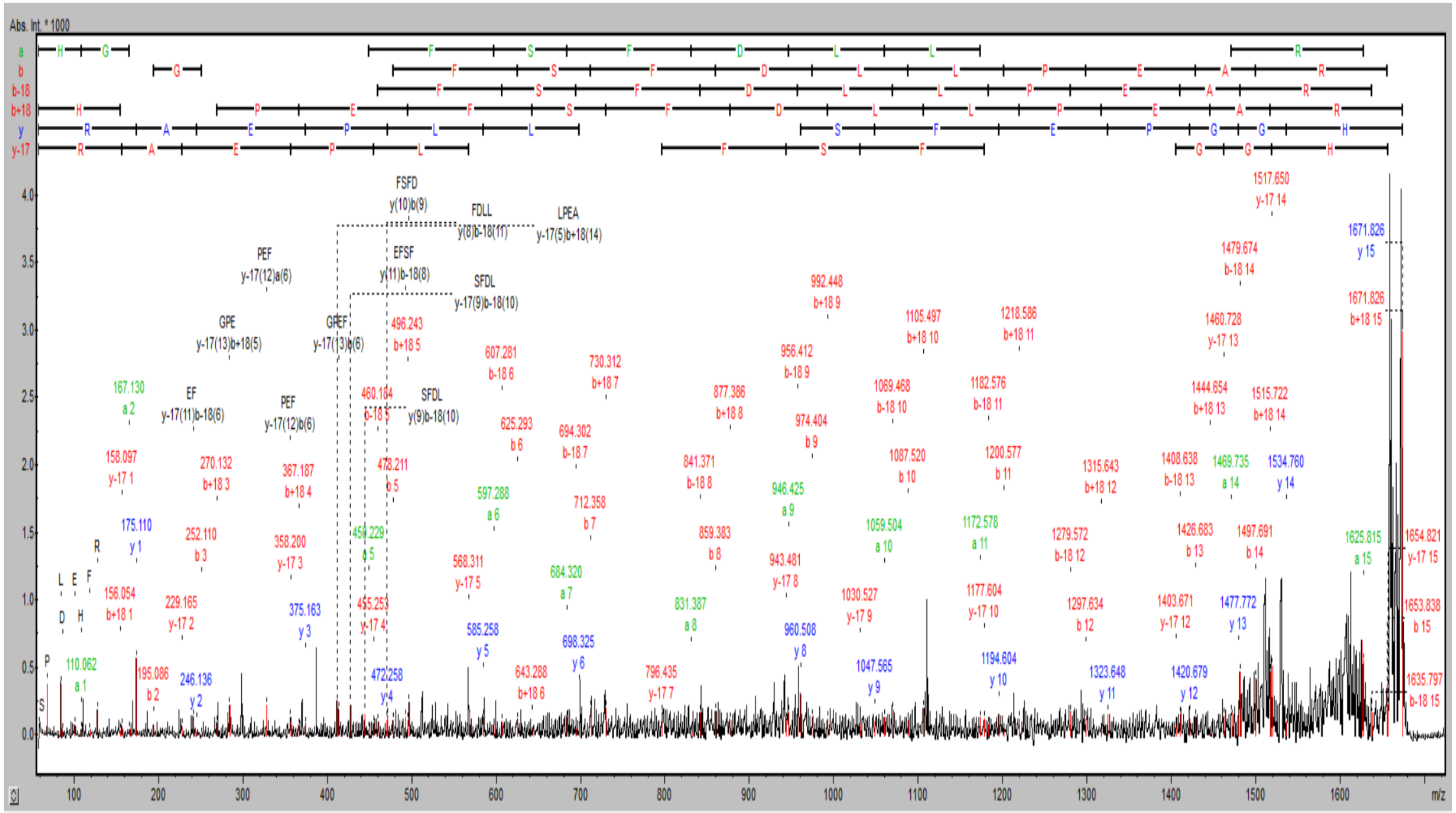

Abs. Int. * 1000
a
b
b-18
b+18
y
y-17
4.0
3.5
3.0
2.5
2.0
1.5
1.0
0.5
0.0
100
200
300
400
500
600
700
800
900
1000
1100
1200
1300
1400
1500
1600
m/z

(C)

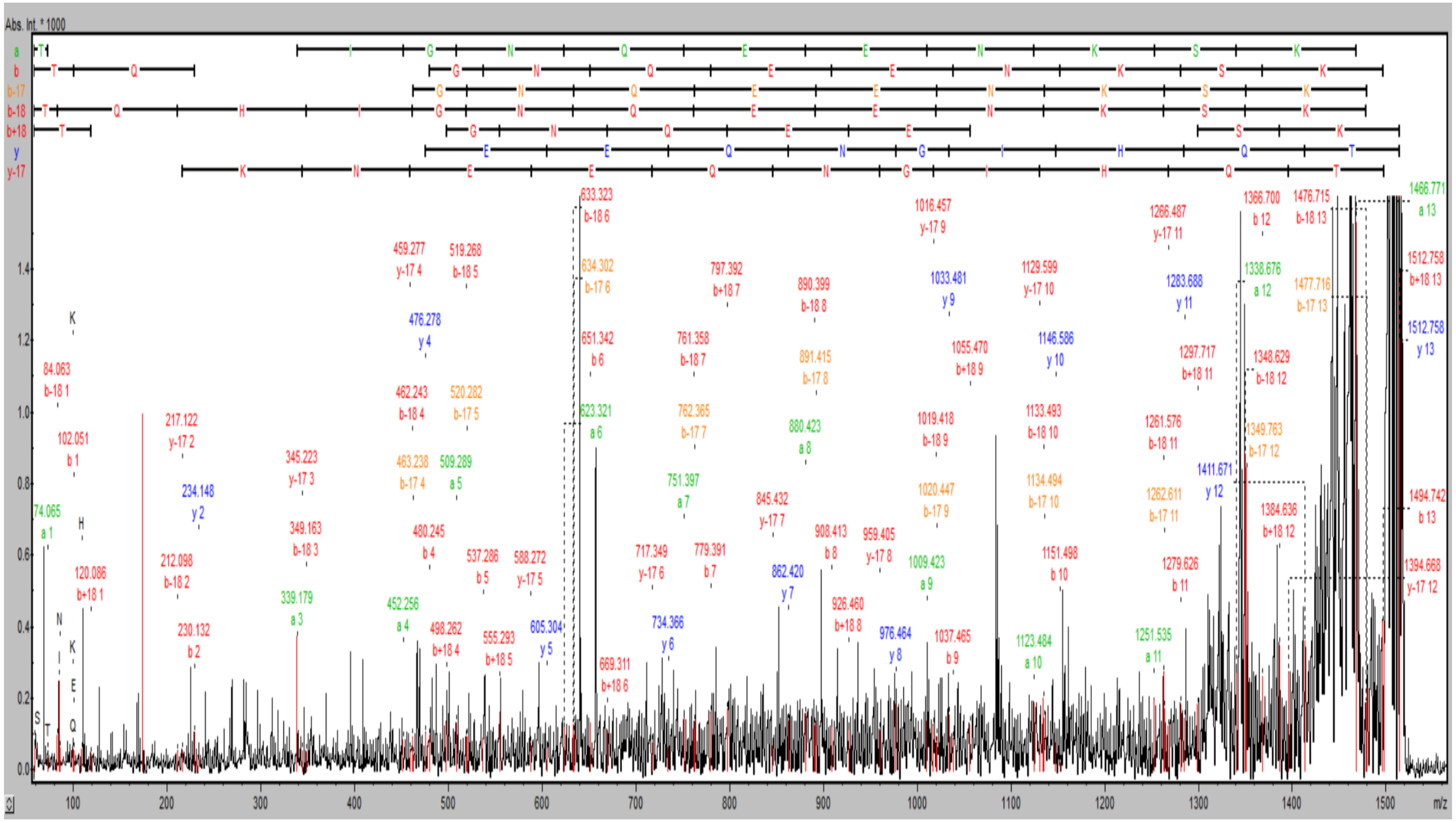

Figure S3. Representative MS/MS spectra of (A) Collagen alpha-1(III) chain, observed mass: 961.49, (B) Interleukin-17 receptor E, observed mass: 1671.8, and (C) Receptor-type tyrosine-protein phosphatase C (CD45), observed mass: 1512.73.
